# 18F-FDG PET/CT for post-cardiac-arrest brain injury: current evidence and future directions

**DOI:** 10.3389/fneur.2026.1875626

**Published:** 2026-08-07

**Authors:** Weijie Cheng, Rui Shao, Xingsheng Wang, Jingfei Yu, Guanghao Yan, Yuehong Guo, Ziren Tang

**Affiliations:** 1 Emergency Medical Center, Beijing Chao-Yang Hospital, Capital Medical University, Beijing, China; 2 Beijing Key Laboratory of Cardiopulmonary-Cerebral Resuscitation Innovation and Translation, Beijing Chao-Yang Hospital, Capital Medical University, Beijing, China; 3 Department of Nuclear Medicine, Beijing Chao-Yang Hospital, Capital Medical University, Beijing, China

**Keywords:** 18F-fluorodeoxyglucose, cardiac arrest, cerebral glucose metabolism, molecular neuroimaging, neuroprognostication, positron emission tomography/computed tomography, post-cardiac-arrest brain injury, targeted temperature management

## Abstract

Post-cardiac-arrest brain injury is a major cause of death and long-term neurological disability after cardiac arrest, yet current prognostic tools provide limited information on regional cerebral metabolism and functional recovery. Fluorine-18 fluorodeoxyglucose positron emission tomography and positron emission tomography/computed tomography (18F-FDG PET and PET/CT) provide a molecular imaging approach for evaluating cerebral glucose metabolism after global ischemia–reperfusion injury. This narrative review summarizes the limited preclinical and clinical evidence on the use of 18F-FDG PET and PET/CT in post-cardiac-arrest brain injury, including animal models, small clinical cohorts, studies of disorders of consciousness, and investigations of neuroprotective interventions such as targeted temperature management. Published studies have documented global and regional abnormalities in cerebral glucose uptake; however, the use of these findings to assess injury severity, monitor treatment response, or predict prognosis remains hypothesis-generating. The evidence base is further limited by small sample sizes, heterogeneous imaging protocols, uncertain optimal timing, cost, radiation exposure, and limited feasibility in critically ill patients. Further prospective studies with standardized acquisition, quantification, and interpretation protocols are needed to determine whether 18F-FDG PET or PET/CT can be integrated into multimodal neuroprognostication and therapeutic monitoring after cardiac arrest.

## Introduction

Cardiac arrest (CA) is associated with substantial mortality and disability, with outcomes varying across regions and healthcare systems (1). In the United States, approximately 350,000–400,000 out-of-hospital cardiac arrests (OHCAs) occur annually, with an overall survival rate of 9.3% and neurologically favorable survival of approximately 8% (2). In the BASIC-OHCA registry in China, the incidence of OHCA was 95.7 per 100,000 person-years; cardiopulmonary resuscitation (CPR) was attempted in 31.85% of cases, and overall and neurologically favorable survival were 1.2 and 0.8%, respectively (3). Globally, survival after OHCA remains low and heterogeneous (4), largely because post-arrest brain injury is a major driver of disability and death (5).

Post-cardiac-arrest brain injury (PCABI) represents a global ischemia–reperfusion injury syndrome. Circulatory arrest interrupts cerebral oxygen and glucose delivery, whereas subsequent reperfusion contributes to mitochondrial dysfunction, inflammation, blood–brain barrier disruption, cerebral edema, and delayed cell death (5–7).

Current tools for assessing the prognosis of brain injury after cardiac arrest include neurological examination, electroencephalography (8), neuroimaging (9, 10) and biomarker testing; however, no single modality provides sufficiently reliable early prognostication, and results should be interpreted within a multimodal framework. Thus, more precise prognostic approaches are needed.

18F-FDG PET or PET/CT has been explored as a tool for evaluating brain injury after cardiac arrest because it can detect abnormalities in cerebral glucose metabolism and, when acquired as PET/CT, provide anatomical localization (11). Preclinical intervention studies further suggest that 18F-FDG PET/CT can detect treatment-related changes in cerebral metabolism after experimental cardiac arrest, although these findings remain hypothesis-generating and do not establish clinical prognostic performance (12).

This review critically summarizes the current evidence, methodological limitations, and research priorities for 18F-FDG PET and PET/CT in PCABI, for which clinical use remains investigational.

## Methods

This article was designed as a structured narrative review and evidence map, rather than a systematic review or meta-analysis. The aim was to critically synthesize the limited evidence on fluorine-18 fluorodeoxyglucose positron emission tomography and positron emission tomography/computed tomography (18F-FDG PET and PET/CT) in post-cardiac-arrest brain injury and to identify methodological limitations and future research priorities.

PubMed/MEDLINE and the Web of Science Core Collection were searched from database inception to May 2026. Only English-language articles were considered eligible. Publication language was verified during full-text assessment when it could not be reliably determined from the database record at the title and abstract screening stage. The search strategy combined terms related to positron emission tomography, PET/CT, FDG-PET, and 18F-FDG with terms related to cardiac arrest, cardiopulmonary arrest, cardiorespiratory arrest, cardiopulmonary resuscitation, return of spontaneous circulation, and the post-resuscitation period. Disease-related terms were intentionally restricted to the cardiac-arrest and post-resuscitation framework to maintain the focus of this review on post-cardiac-arrest brain injury. The complete search strategies are provided in Appendix 1.

Studies were eligible if they were original human or animal investigations involving cardiac arrest, cardiopulmonary arrest, cardiorespiratory arrest, cardiopulmonary resuscitation, return of spontaneous circulation (ROSC), or the post-resuscitation period; used positron emission tomography or positron emission tomography/computed tomography with a tracer protocol that included 18F-FDG; and evaluated brain injury, cerebral glucose metabolism, regional metabolic abnormalities, neurological outcomes, neuroprognostication, targeted temperature management, neuroprotective interventions, or mechanisms relevant to post-cardiac-arrest brain injury. Studies were excluded if they were unrelated to cardiac arrest or the post-resuscitation period, did not use PET or PET/CT, did not include 18F-FDG, did not assess brain injury or relevant neurological or metabolic outcomes, or were reviews, guidelines, editorials, letters, conference abstracts, or other non-original articles. Relevant reviews and guidelines were used only for background and contextual information.

Two reviewers independently screened titles and abstracts in parallel, without consultation during the initial screening process. Disagreements were resolved through discussion; if consensus could not be reached, a third expert was invited to adjudicate.

**Figure 1:**
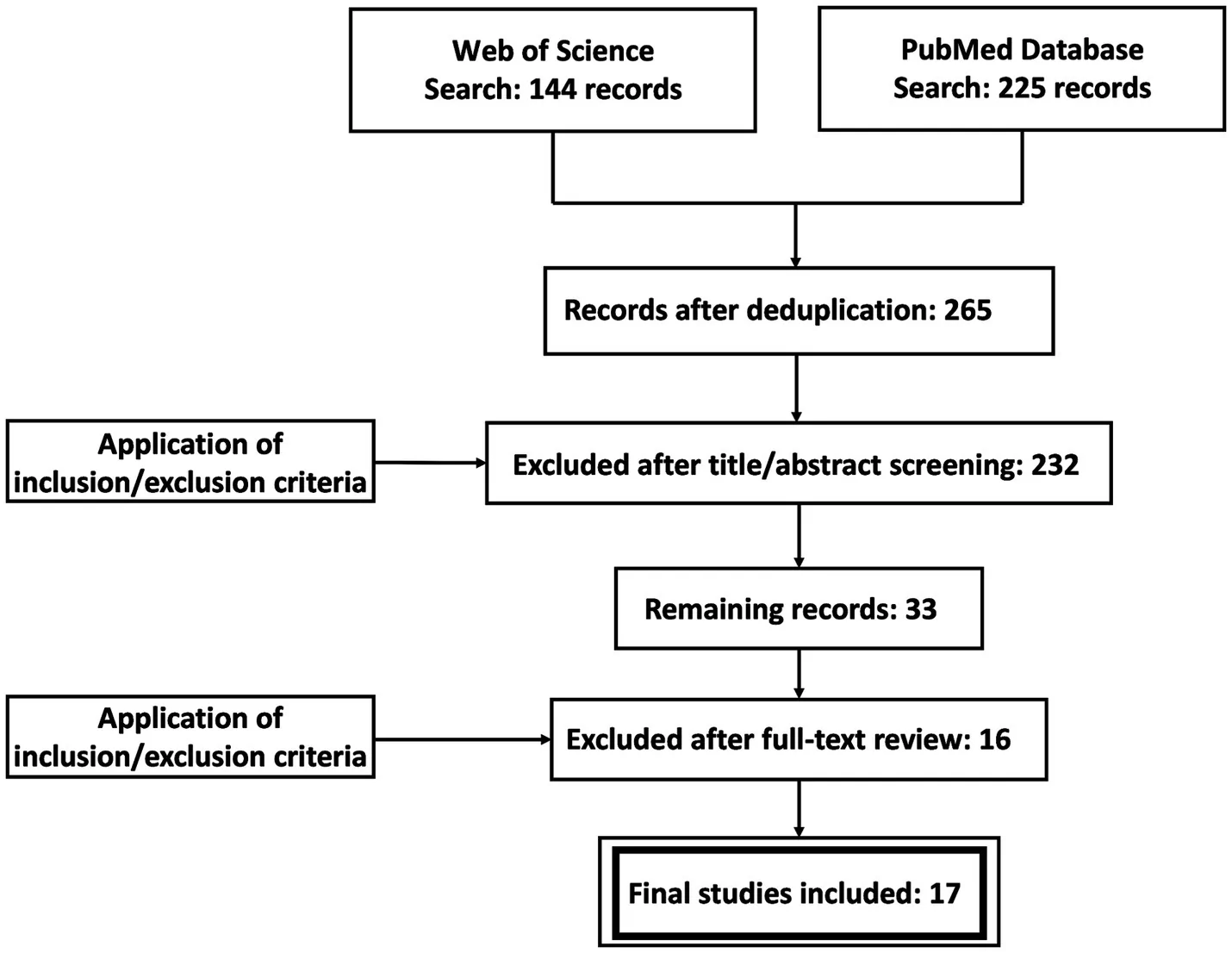
PRISMA-ScR-style flow diagram of study identification, screening, eligibility assessment, and inclusion.

The database search identified 369 records, including 225 from PubMed/MEDLINE and 144 from the Web of Science Core Collection. After removal of 104 duplicates, 265 records underwent title and abstract screening. Of these, 232 records were excluded during title and abstract screening, and 33 articles underwent full-text assessment for eligibility. Sixteen articles were excluded during full-text assessment after application of the predefined eligibility criteria. Seventeen studies were included in the evidence table. Additional adjacent studies that did not meet the predefined criteria for inclusion in the evidence table were discussed narratively where relevant. The study-selection process is summarized in Figure 1.

For each included study, key information was extracted on study design, population or model, PET methodology, imaging timing, outcome assessment, and main findings. To improve the interpretability of the small and heterogeneous evidence base, studies were additionally summarized according to evidence source, sample-size category, and key methodological limitations. No protocol was registered, and no formal meta-analysis, risk-of-bias assessment, evidence-certainty assessment, or quantitative synthesis was performed. Therefore, these descriptors should be interpreted as a structured narrative appraisal rather than as formal Grading of Recommendations Assessment, Development and Evaluation (GRADE) certainty ratings or risk-of-bias scores.

## Post-cardiac-arrest brain injury: pathophysiology and current assessment

### Pathophysiological mechanisms

A recent International Liaison Committee on Resuscitation (ILCOR) scientific statement conceptualized PCABI as four distinct but overlapping phases: ischemic depolarization, reperfusion repolarization, dysregulation, and recovery and repair. Ischemic depolarization reflects the cessation of cerebral blood flow, resulting in energy failure, ionic imbalance, calcium overload, and acidosis. Reperfusion repolarization is characterized by partial restoration of blood flow, accompanied by persistent mitochondrial dysfunction and oxidative stress. The dysregulation phase involves inflammation, blood–brain barrier disruption, cerebral edema, metabolic failure, and delayed cell death. During recovery and repair, neuroplasticity and synaptic remodeling may occur, although the balance between recovery and secondary injury remains variable (6).

These phases provide a biological framework for interpreting temporal PET patterns. During ischemic depolarization, oxygen and glucose delivery cease, and FDG uptake may be globally reduced if the tracer is administered during severe hypoperfusion. Early reperfusion may produce heterogeneous uptake because macrovascular blood flow can recover while mitochondrial function, microcirculation, and synaptic activity remain impaired. During the dysregulation phase, inflammation, edema, seizures, hyperglycemia, and sedative exposure may either increase or suppress regional FDG signals, making isolated SUV thresholds unreliable. In the recovery-and-repair phase, persistent cortical or subcortical hypometabolism may reflect loss of viable neuronal networks, whereas preservation or restoration of network-level metabolism may be more closely related to functional outcome than whole-brain uptake alone (13, 14).

### Limitations of current assessment tools

Neurologic examination, electroencephalography (EEG), somatosensory evoked potentials (SSEP), transcranial Doppler (TCD), computed tomography (CT), magnetic resonance imaging (MRI), and neuron-specific enolase (NSE) are used to assess PCABI, but each has prognostic limitations. Clinical examination may be confounded by sedation, neuromuscular blockade, and temperature (15, 16). EEG findings require multimodal interpretation because sedation complicates assessment, and SSEP have limited sensitivity, risking false-negative results in severe brain injury (17); TCD is operator dependent, requires an adequate acoustic window, is influenced by pH, heart rate, and CO_2_, and reflects macrovascular flow rather than microcirculatory perfusion (18); CT may miss early subtle PCABI because of limited soft-tissue sensitivity; interpretation using gray–white matter ratios (GWRs) is further limited by the lack of standardized thresholds (19); MRI is more sensitive than CT for ischemic injury, but apparent diffusion coefficient (ADC) thresholds vary across methods, regions, and timing, limiting clinical consistency (20); NSE lacks brain specificity; hemolysis and other noncerebral sources may falsely elevate levels, limiting prognostic accuracy (21). The optimal timing of imaging after cardiac arrest remains uncertain, underscoring the need for more reliable assessment strategies. 18F-FDG PET or PET/CT may help address important limitations of current prognostic tools.

Accordingly, 18F-FDG PET or PET/CT should be evaluated against established neuroprognostication tools rather than regarded as a replacement for them. Current guidelines emphasize delayed, multimodal prognostication based on clinical examination combined with EEG, SSEP, CT or MRI, and serum biomarkers, while accounting for confounders such as sedation, hypothermia, metabolic derangements, and time from ROSC (22). 18F-FDG PET provides regional metabolic information not captured by CT or routine MRI, whereas PET/CT additionally enables CT-based anatomical co-localization when hybrid PET/CT acquisition is performed. However, its use in PCABI remains investigational because validated PET thresholds, optimal imaging timing, and incremental predictive value beyond existing tools have not been established.

## 18F-FDG PET and PET/CT: principles, quantification, and clinical use

### Principles of 18F-FDG PET and PET/CT in PCABI

**Figure 2:**
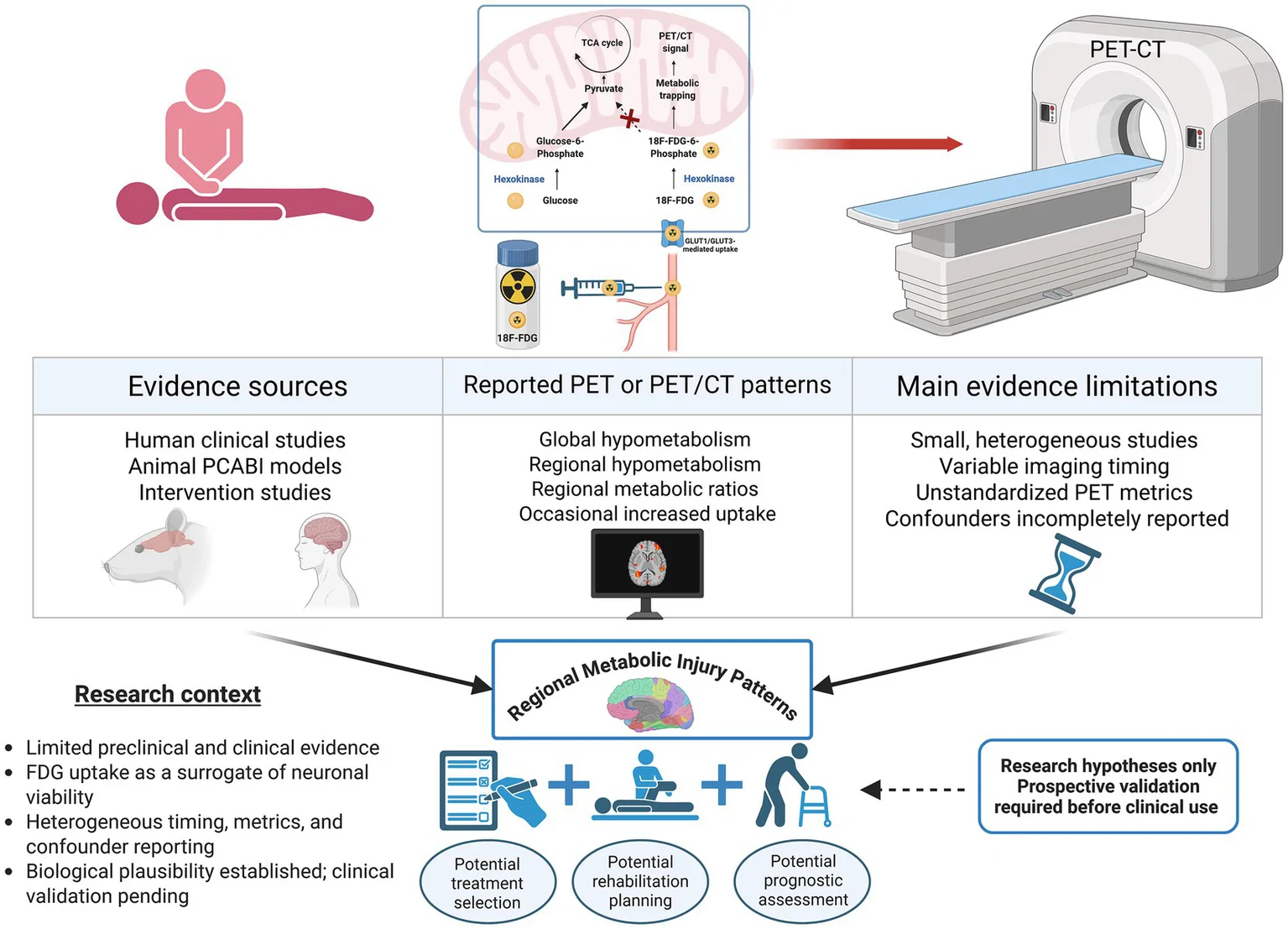
Conceptual framework and evidence map for 18F-FDG PET/CT in post-cardiac-arrest brain injury. The figure summarizes the biological basis of 18F-FDG uptake, evidence sources, reported PET or PET/CT patterns, main evidence limitations, and regional metabolic injury patterns after cardiac arrest. The upper panel illustrates the relationship between native glucose metabolism and 18F-FDG trapping. Treatment selection, rehabilitation planning, and prognostic assessment represent potential future applications requiring prospective multicenter validation, not current clinical recommendations. Created with BioRender.com.

PET provides functional metabolic imaging, whereas PET/CT combines PET-derived metabolic information with CT-based anatomical localization (23). Figure 2 presents a schematic conceptual framework and evidence map for interpreting 18F-FDG PET and PET/CT findings in post-cardiac-arrest brain injury. The figure summarizes the biological basis of FDG uptake, available evidence sources, reported PET or PET/CT patterns, key limitations of the evidence, and the current gap between biological plausibility and clinical translation.

### Investigational role of 18F-FDG PET and PET/CT in PCABI

18F-FDG PET or PET/CT has been investigated in a limited number of studies relevant to PCABI because it provides metabolic information related to hypoxic–ischemic brain injury and, when acquired as PET/CT, anatomical localization. By identifying regional abnormalities in glucose metabolism, 18F-FDG PET or PET/CT may help characterize the extent and distribution of injury in vulnerable regions such as the cortex, hippocampus, and thalamus. These findings support the biological plausibility of 18F-FDG PET or PET/CT in PCABI but do not establish its clinical prognostic or management utility (12, 13, 24).

### PET quantification and interpretation

The reviewed studies used several non-equivalent PET metrics. Standardized uptake value (SUV) and maximum standardized uptake value (SUVmax) are semiquantitative measures influenced by injected activity, body size normalization, uptake time, scanner reconstruction, blood glucose level, and partial-volume effects. Standardized uptake value ratio (SUVR) normalizes uptake to a reference region, which may improve interscan comparability but can introduce bias when the reference region is injured, temperature-sensitive, or biologically unstable. In addition, partial-volume effects and injury involving commonly used reference regions, such as the cerebellum, pons, or whole brain, may bias SUV, SUVmax, and SUVR estimates, thereby limiting the transferability of thresholds across studies (25). Cerebral metabolic rate of glucose (CMRglc) is a more physiologically specific measure of cerebral glucose metabolism but generally requires kinetic modeling and an arterial or image-derived input function, which can be difficult to obtain in unstable intensive care unit patients. Future studies of post-cardiac-arrest brain injury should therefore report acquisition timing, uptake duration, blood glucose level, temperature status, sedation exposure, reconstruction method, region-of-interest definition, and whether analyses are based on SUV, SUVR, voxel-based statistics, or CMRglc (25, 26).

### Bidirectional FDG signals: neuronal metabolism versus neuroinflammation

The direction of FDG signal changes after cardiac arrest should be interpreted in relation to imaging timing, brain region, and cellular source. Reduced FDG uptake may reflect impaired perfusion, mitochondrial dysfunction, loss of synaptic activity, neuronal death, or network disconnection. Conversely, increased FDG uptake does not necessarily indicate preserved neuronal viability, because activated microglia, astrocytes, seizures, and reperfusion-related inflammatory stress may also increase glucose utilization. Therefore, the increased uptake reported by Zhang et al. and the TLR2-dependent metabolic response described by Bajorat et al. may reflect injury-related neuroinflammatory activation rather than simple neuronal recovery. This bidirectional biological response limits the reliability of isolated SUV or SUVmax thresholds. Accordingly, 18F-FDG PET or PET/CT findings should be interpreted in conjunction with timing after ROSC, temperature, glucose level, sedation, seizure activity, perfusion status, and multimodal neurological assessment (13, 14).

### Evidence base for 18F-FDG PET and PET/CT after cardiac arrest

Table 1 presents a condensed per-study evidence summary that retains all 17 included studies, separates human and animal evidence, highlights sample sizes, provides descriptive evidence categories, and summarizes the main methodological limitation or risk-of-bias concern for each study. Figure 2 functions as a schematic evidence map, summarizing evidence sources, reported PET or PET/CT patterns, key limitations of the evidence, and the current gap between biological plausibility and clinical translation.

**Table 1:** Condensed per-study evidence table of published 18F-FDG PET and PET/CT studies relevant to post-cardiac-arrest brain injury.

Evidence category	Study	*n*/size	Population/model	Timing	Imaging modality and PET metric	Evidence-supported finding	Main limitation/risk-of-bias concern
Human clinical CA-specific	DeVolder et al., 1990 (34)	12 (very small)	Postanoxic syndrome after cardiac arrest	Postanoxic stage; interval NR	18F-FDG PET; regional CMRglc vs. normal values	Global hypometabolism was observed, greatest in vegetative patients, with parieto-occipital/border-zone changes.	Historical small cohort; timing not reported; no contemporary multimodal adjustment.
Human clinical CA-specific/contextual	Rudolf et al., 1999 (38)	24 (small)	AVS/PVS after cardiorespiratory arrest	6–165 days; median 33 days	18F-FDG PET; regional CMRglc; standardized ROIs	Cortical CMRglc was reduced, especially in PVS; PET did not clearly distinguish death from persistent VS.	Delayed and variable imaging; heterogeneous disorders of consciousness; limited outcome discrimination.
Large-animal experimental	Thorngren-Jerneck et al., 2001 (29)	16 (very small)	Near-term lambs after asphyxia-induced arrest	4 h after resuscitation	PET; global CMRglc	Global CMRglc was reduced after asphyxia-induced arrest, indicating early hypoxic–ischemic injury.	Neonatal lamb model; limited direct adult PCABI translation.
Human clinical CA-specific	Schaafsma et al., 2003 (35)	8 (very small)	Severe post-hypoxic encephalopathy after cardiac arrest	Day 1 post-resuscitation	PET; regional cerebral glucose metabolism and perfusion	FDG uptake was markedly depressed; PET added limited prognostic value in a uniformly poor-outcome cohort.	Very small, uniformly poor-outcome cohort; limited prognostic range.
Human clinical TTM	Nakamura et al., 2008 (45)	7 (very small)	Comatose patients receiving hypothermia after CPA	End of 72 h hypothermia	PET; FDG uptake, OEF, and lactate	Poor outcomes showed reduced FDG uptake; better outcomes showed higher oxygen extraction during hypothermia.	Very small cohort; imaging during hypothermia; incomplete confounder reporting.
Large-animal intervention	Zhang et al., 2014 (33)	34 (small)	Pig VF arrest; Shenfu vs. saline/sham	24–48 h after ROSC	18F-FDG PET/CT acquisition; regional SUVmax	Shenfu attenuated cerebral SUVmax decline and was associated with better neurological and mitochondrial measures.	Preclinical intervention model; protocol- and treatment-specific results.
Large-animal serial model	Li et al., 2015 (28)	6 (very small)	Beagle VF arrest with CPR/ROSC	Baseline, 4, 24, 48 h	Dynamic 18F-FDG PET/CT acquisition; CMRglc and hexokinase I/II	Cerebral metabolism fell at 4 h and remained below baseline at 24–48 h.	Extremely small sample; animal model; limited generalizability.
Large-animal comparative model	Zhang et al., 2015 (27)	44 (small)	Pig VF vs. asphyxial cardiac arrest	24 h after ROSC	18F-FDG PET/CT acquisition; regional SUVmax	Asphyxial arrest caused lower SUVmax and worse neurological and survival outcomes than VF arrest.	Preclinical model comparison; no validated clinical threshold.
Small-animal regional model	Putzu et al., 2018 (30)	18 (very small)	Rat cardiac arrest/resuscitation model	Post-resuscitation; exact time NR	18F-FDG PET/CT acquisition and autoradiography; regional glucose metabolism	Regional metabolic differences after CA supported region-specific PET assessment.	Small sample; exact timing not reported; PET/autoradiography translation issues.
Small-animal prognostic model	Kim et al., 2019 (31)	18 (very small)	Rat post-cardiac-arrest syndrome model	Baseline and 3 h after CA/resuscitation	18F-FDG PET/CT acquisition; PET-derived brain metabolic ratio	Forebrain-to-hindbrain ratio predicted good neurological outcome, supporting regional metabolic ratios as early indicators.	Animal cognitive outcome; regional ratio unvalidated clinically.
Human clinical contextual	Scheibe et al., 2020 (39)	72 (moderate)	Adults with HIE after cardiac arrest	Delayed phase; timing variable/NR	18F-FDG PET/CT acquisition; regional metabolic pattern	Basal ganglia hypometabolism localized post-hypoxic movement-disorder lesions.	Retrospective design; delayed/variable timing; movement-disorder-focused outcome.
Small-animal mechanistic	Zhang et al., 2021 (13)	25 (small)	Mouse KCl-induced cardiac arrest/CPR	72 h after arrest	18F-FDG PET/CT acquisition; regional cerebral glucose metabolism	FDG uptake increased, especially in brainstem regions, and correlated with worse injury.	Mouse KCl model; increased uptake may reflect inflammation or seizures.
Small-animal mechanistic	Bajorat et al., 2022 (14)	27 (small)	WT and TLR2-deficient mice after CA/CPR	~90 min after resuscitation	18F-FDG PET/CT acquisition; regional cerebral glucose metabolism	Uptake increased in WT but not TLR2-deficient mice, implicating TLR2-related inflammation.	Genetic mouse model; very early imaging; limited clinical translation.
Large-animal/rabbit intervention	Li et al., 2022 (12)	15 (very small)	Rabbit CA; hydrogen vs. control/sham	Early post-resuscitation time points	18F-FDG PET/CT acquisition; cerebral glucose metabolism	Hydrogen altered FDG uptake and was associated with less neurological and histological injury.	Preclinical intervention model; small sample; timing heterogeneity.
Small-animal intervention	Peng et al., 2022 (32)	43 (small)	Mouse CA/CPR with ketogenic diet	After CA/CPR; exact time NR	18F-FDG PET/CT acquisition; regional cerebral glucose metabolism	Ketogenic diet improved recovery and reduced apoptosis, linked to antioxidant metabolic pathways.	Exact PET timing NR; intervention-specific exploratory mechanism.
Human clinical exploratory ratio	Rossato et al., 2022 (11)	37 (small)	Adults with hypoxic/anoxic injury after circulatory arrest	Early rehabilitation phase	18F-FDG PET/CT acquisition; cerebellar vermis-to-hemisphere metabolic ratio	V/C ratio was associated with recovery; the 1.5 cutoff remains exploratory.	Single-center retrospective cohort; unvalidated threshold; not stand-alone prognostication.
Small-animal TTM model	Kim et al., 2024 (46)	21 (small)	Rat asphyxial arrest; hypothermia vs. control	After 8 h cooling before PET	18F-FDG PET/CT acquisition; regional FDG uptake / SUV-based metrics	Hypothermia did not improve overall survival/SUVR, but nonsurvivors showed higher cortical SUVR.	Rat TTM model; limited survival effect; reference-region dependence.

Evidence categories are descriptive and were used to distinguish human clinical, contextual, large-animal, and small-animal evidence. These categories should not be interpreted as formal GRADE certainty ratings. Sample-size categories were defined descriptively as very small (*n* < 20), small (*n* = 20–49), and moderate (*n* ≥ 50). The final column summarizes the main methodological limitation or risk-of-bias concern relevant to interpretation.

Overall, these studies support biological plausibility but do not yet establish clinical readiness. Most preclinical experiments used small animal samples and varied in arrest mechanisms, imaging time points, and quantification methods. Human evidence is dominated by small retrospective cohorts, delayed imaging, heterogeneous disorders of consciousness, and inconsistent outcome definitions. Key confounders, including sedation, glycemic status, temperature management, seizure activity, and reference-region injury, were rarely reported in sufficient detail to support cross-study thresholds. Thus, the current evidence supports hypothesis generation and study design rather than stand-alone clinical prognostication.

### Animal experimentation

Research on 18F-FDG PET or PET/CT after cardiac arrest remains preliminary. Current evidence is limited to animal studies, small clinical cohorts, and indirect findings, and systematic clinical application is not established.

### Glucose uptake and metabolism

In animal models, 18F-FDG PET or PET/CT has been used to characterize ischemia–reperfusion-related alterations in cerebral glucose metabolism; however, these studies do not establish clinical prognostic performance. In a mouse model, whole-brain SUV at 72 h was higher after cardiac arrest than after sham operation or in controls, with the greatest increases in the midbrain, pons, and medulla. Biodistribution analysis confirmed greater tracer uptake, suggesting increased metabolic demand after severe neurologic injury (13). At 24 h after resuscitation, SUVmax was lower after asphyxial cardiac arrest than after ventricular fibrillation cardiac arrest, indicating more severe metabolic impairment. These PET/CT findings paralleled worse neurologic deficit scores and higher serum NSE and S100β levels, demonstrating distinct metabolic injury patterns between arrest mechanisms (27). In Beagles after cardiac arrest, 18F-FDG PET/CT showed that cerebral glucose metabolism fell sharply 4 h after return of spontaneous circulation and remained depressed for 48 h, paralleling hexokinase I and II expression and supporting detection of hyperacute ischemic injury (28).

Earlier animal studies also indicate that 18F-FDG PET can detect early cerebral metabolic depression after hypoxic–ischemic arrest. In near-term fetal lambs subjected to asphyxia-induced cardiac arrest and standardized resuscitation, the global cerebral metabolic rate of glucose was significantly reduced at 4 h, indicating early post-resuscitation hypoxic–ischemic cerebral dysfunction (29). In a rat cardiac-arrest/resuscitation model, combined 18F-FDG PET and autoradiography revealed region-dependent changes in cerebral glucose metabolism after resuscitation, supporting regional rather than whole-brain interpretation (30). Kim et al. further showed that, in a rat model of post-cardiac-arrest syndrome, early 18F-FDG PET performed 3 h after resuscitation could stratify subsequent neurocognitive outcomes, with the forebrain-to-hindbrain metabolic ratio serving as a potential early prognostic index (31).

### Validation of neuroprotective effects

18F-FDG PET/CT has been used experimentally to evaluate neuroprotective effects by detecting treatment-related metabolic alterations. In a mouse cardiac arrest model, a ketogenic diet improved neurologic function and reduced neuronal apoptosis despite unchanged cerebral glucose uptake. Integrated metabolic analyses suggested that these effects may be mediated, at least in part, by reduced oxidative stress through activation of antioxidant defenses and the pentose phosphate pathway. These findings support mechanistic interpretation rather than clinical treatment monitoring (32). In a rabbit model, hydrogen treatment was associated with significant SUVmax changes in multiple brain regions at 2 and 24 h after resuscitation, indicating that 18F-FDG PET/CT is sensitive to early metabolic alterations. These findings were accompanied by lower neurological deficit scores, less neuronal injury, and favorable changes in UCH-L1 and NSE, although they remain preclinical and hypothesis-generating (12). In minipigs after cardiac arrest, Shenfu injection was associated with a smaller reduction in regional SUVmax at 48 h than saline, consistent with improved mitochondrial function and neurologic scores. These findings support further testing of PET-based metabolic readouts (33).

### Immune and oxidative stress-related metabolism

18F-FDG PET/CT has been used to explore metabolic alterations associated with immune activation and oxidative stress, thereby providing preclinical mechanistic insight into post-resuscitation brain injury. Bajorat et al. used 18F-FDG PET/CT to compare cerebral glucose metabolism before and after cardiac arrest and cardiopulmonary resuscitation in wild-type and Toll-like receptor 2 (TLR2)-deficient mice. They found that wild-type mice had a 25.6% increase in whole-brain mean standardized uptake value (SUVmean) approximately 90 min after resuscitation, whereas metabolism in TLR2-deficient mice was not significantly altered and baseline glucose uptake was 34.6% higher in TLR2-deficient mice. These findings suggest that 18F-FDG PET/CT can detect TLR2-related metabolic differences after cardiac arrest and cardiopulmonary resuscitation (14).

Taken together, animal studies support the biological plausibility of 18F-FDG PET or PET/CT for detecting post-resuscitation metabolic injury. Clinical translation remains limited by heterogeneity in arrest mechanisms, anesthesia protocols, temperature control, tracer-injection timing, imaging time points, and neurological scoring systems.

## Clinical studies

### Glucose uptake and metabolism

Earlier clinical studies provided foundational but heterogeneous evidence of post-anoxic cerebral metabolic impairment. Using 18F-FDG PET, DeVolder et al. reported marked global cerebral hypometabolism in patients with post-anoxic syndrome after cardiac arrest, which was most severe in patients in a vegetative state and was accompanied by regional abnormalities in the parieto-occipital and arterial border-zone regions (34). Schaafsma et al. performed PET on day 1 after resuscitation in patients with severe post-hypoxic encephalopathy caused by cardiac arrest and found markedly reduced 18F-FDG uptake. However, PET did not provide substantial prognostic information beyond serial neurological assessment in this cohort, in which outcomes were uniformly poor (35).

As historical contextual human evidence, Tommasino et al. reported reduced regional cerebral glucose metabolism on 18F-FDG PET in comatose patients and patients in a vegetative state. Because the cohort included mixed etiologies and cardiac-arrest-specific results could not be separately extracted, this study was discussed as contextual evidence rather than included in the evidence table (36).

Bertagnoni et al. further evaluated 18F-FDG PET in patients with hypoxic–ischemic encephalopathy after cardiac arrest or respiratory failure, suggesting that PET-derived metabolic patterns may provide information relevant to outcome and rehabilitation prognostication. These findings should be interpreted as contextual support for biological plausibility, rather than as cardiac-arrest-specific prognostic validation. Because the cohort was etiologically mixed, this study was considered contextual clinical evidence rather than a cardiac-arrest-specific validation study (37).

Rudolf et al. used 18F-FDG PET to examine 24 patients in an acute vegetative state (AVS, < 1 month; *n* = 11) or persistent vegetative state (PVS, > 1 month; *n* = 13) after cardiorespiratory arrest. They found that the whole-brain cerebral metabolic rate of glucose (CMRglc) was significantly lower than that of age-matched controls, particularly in cortical regions, whereas the reduction was less pronounced in subcortical structures. Cortical CMRglc was significantly lower in patients with PVS than in those with AVS (*p* < 0.05, except in the frontal lobe), reflecting progressive loss of residual cortical function after hypoxic injury and consistent with Wallerian and transsynaptic degeneration observed in neuropathology. Although 18F-FDG PET demonstrated the dynamic course of post-hypoxic metabolic alterations, differences in metabolic rate did not significantly distinguish patients who died from those who survived (38).

### Locating metabolic abnormalities in specific brain regions

18F-FDG PET/CT has been reported to identify region-specific metabolic abnormalities associated with neurological impairment. In a retrospective study of 72 patients with hypoxic–ischemic encephalopathy after cardiac arrest between 2007 and 2018, Scheibe et al. reported that 19 patients developed post-hypoxic dyskinesia. In these patients, 18F-FDG PET/CT revealed hypometabolism in the basal ganglia, corresponding to hyperintensity on T1-weighted MRI, suggesting that basal ganglia injury is a major neuroanatomical correlate of post-hypoxic movement disorders. The study suggested that 18F-FDG PET/CT may help localize injury patterns and generate hypotheses for symptom-directed management, including symptom control with levomepromazine or intrathecal baclofen. Remission of dyskinesia was observed in 58% of patients (39).

### Metabolic ratios or regional variations

Regional metabolic ratios may provide exploratory prognostic information, although the current evidence remains preliminary. In a single-center retrospective study of 37 patients with hypoxic/anoxic brain injury after cardiocirculatory arrest, Rossato et al. reported that the cerebellar vermis-to-hemisphere metabolic ratio (V/C ratio) was associated with changes in clinical assessment scales, including the FIM, LCFS, GOS, and CRS-R. A V/C ratio ≥ 1.5 was proposed in that cohort as a potential marker of poorer recovery. However, this cut-off was derived from a single cohort, has not been externally validated, and should not be used as a stand-alone prognostic threshold. These findings suggest that regional metabolic ratios are hypothesis-generating and require validation in larger prospective cohorts (11).

Human studies provide clinically relevant signals but remain limited by small retrospective cohorts, delayed or variable imaging, heterogeneous populations with disorders of consciousness, and inconsistent reporting of sedation, glycemic status, seizure activity, temperature management, and outcome definitions.

### Regional vulnerability and network-level interpretation

Regional vulnerability is central to interpreting PET findings in PCABI. Global ischemia does not affect all brain regions uniformly. The cortex, hippocampus, thalamus, basal ganglia, cerebellum, and brainstem may exhibit distinct metabolic trajectories depending on arrest mechanism, reperfusion quality, seizure burden, and time from ROSC. In the reviewed studies, poor outcomes were associated with diffuse cortical hypometabolism, altered forebrain-to-hindbrain metabolic relationships, basal ganglia abnormalities in post-hypoxic movement disorders, and cerebellar vermis-to-hemisphere ratios in post-anoxic disorders of consciousness (31, 40, 41). These findings are hypothesis-generating and suggest that metabolic ratios and network-level patterns may be more informative than isolated regional maximum standardized uptake value (SUVmax). However, their interpretation requires standardized region-of-interest (ROI) definitions and independent validation.

### Targeted temperature management (TTM)

TTM is an established component of post-resuscitation care, but its effects on cerebral metabolism are complex and context-dependent (42–44). The use of 18F-FDG PET or PET/CT to assess TTM after cardiac arrest remains exploratory. Therefore, PET-based assessment of cerebral metabolism during TTM should be framed as a research hypothesis rather than as an established tool for optimizing temperature management (45, 46). Most studies of 18F-FDG PET or PET/CT in this specific scenario remain animal experiments or small clinical studies, and no broad consensus on clinical applications has yet emerged.

The TTM literature also changes how 18F-FDG PET or PET/CT should be interpreted. The TTM2 trial showed that targeted hypothermia at 33 °C did not reduce 6-month mortality or poor functional outcome compared with targeted normothermia and active fever treatment; however, hypothermia was associated with a higher incidence of arrhythmias causing hemodynamic compromise (47). Accordingly, the 2022 European Resuscitation Council–European Society of Intensive Care Medicine (ERC–ESICM) temperature-control guideline emphasized continuous core-temperature monitoring and prevention of fever above 37.7 °C for at least 72 h, while noting insufficient evidence to recommend for or against temperature control at 32–36 °C in all comatose adults after cardiac arrest (48). The 2025 ERC–ESICM post-resuscitation guidelines continue to position temperature control as one component of comprehensive intensive care unit (ICU) management, alongside hemodynamic optimization, ventilation, seizure management, prognostication, rehabilitation, and assessment of long-term outcomes (22).

For PET interpretation, hypothermia should be considered a biological confounder rather than merely a treatment label. Cooling may reduce cerebral metabolic demand and alter hemodynamics, glucose transport, enzymatic phosphorylation, shivering, insulin use, sedative exposure, and tracer kinetics. Therefore, a scan acquired during active cooling may differ from one obtained during normothermia or after rewarming, even when tissue viability remains unchanged. Future targeted temperature management–PET studies should prespecify whether FDG is administered during cooling, temperature maintenance, rewarming, or normothermia. They should also report core temperature, glucose level, insulin use, sedative exposure, vasopressor support, and seizure activity, and should avoid interpreting treatment-related SUV changes without accounting for these variables (45).

Mechanistic evidence from a global cerebral ischemia model further suggests that hypothermia may selectively preserve the anterior forebrain mesocircuit, supporting the concept that temperature management can modify region- and network-level vulnerability after global ischemic injury. Because this study did not use a cardiac-arrest/resuscitation 18F-FDG PET or PET/CT, it was discussed as mechanistic context rather than included in Table 1 (49).

18F-FDG PET or PET/CT has been used in preclinical and small clinical studies to explore the neuroprotective effects of targeted temperature management by detecting treatment-related metabolic alterations. In a rat model, hypothermia did not improve overall survival or cortical uptake overall, but in nonsurvivors it was associated with higher cortical SUVR (pons), suggesting a possible metabolic benefit in severe ischemic brain injury (46). Existing clinical studies remain too small to determine whether PET can reliably monitor cerebral metabolism during targeted temperature management after cardiac arrest. In a small cohort, reduced FDG uptake was observed in patients with poor outcomes, whereas higher oxygen extraction fraction was seen in those with better outcomes, suggesting potential value for assessing treatment effects and prognostic differences (45).

These practical barriers, including cost, radiation exposure, transport risks, and monitoring requirements, are discussed in greater detail below.

## Current limitations and research priorities

### Potential research value of 18F-FDG PET and PET/CT in PCABI

PCABI is a major cause of death and disability in comatose survivors after cardiac arrest, and outcome heterogeneity across regions and healthcare systems underscores the need for reproducible assessment strategies (22, 50). Imaging techniques are therefore commonly used to assess neurological prognosis and tissue injury. Compared with CT and MRI, 18F-FDG PET or PET/CT has been less extensively evaluated in this setting (51). Existing clinical and animal studies remain limited by small sample sizes, heterogeneous methods, and uncertain reproducibility (11, 32). Therefore, its current value lies primarily in generating research questions about regional cerebral metabolism, rather than in guiding clinical decision-making.

The research value of 18F-FDG PET or PET/CT lies in its ability to provide regional metabolic information, whose incremental value should be tested against structural imaging, electrophysiology, serum biomarkers, and clinical assessment.

### Alternative tracers and multimodal PET strategies for future research

A forward-looking PET strategy should not rely on FDG alone. FDG reflects glucose utilization and synaptic activity but cannot distinguish reduced neuronal viability from the effects of sedation, hypothermia, hyperglycemia, seizures, or perfusion-limited tracer delivery. Perfusion tracers, such as oxygen-15-labeled water (15O-H2O), may help differentiate metabolic failure from blood-flow limitation. Translocator protein (TSPO) tracers could be used to assess neuroinflammation and microglial activation, whereas flumazenil-based tracers may provide information on neuronal receptor integrity (52–55). None of these tracers is ready for routine use in post-cardiac-arrest brain injury care. They are best framed as exploratory tools for future mechanistic studies.

### Research priorities before clinical translation

Future research should determine whether 18F-FDG PET or PET/CT provides reproducible information and incremental value in PCABI. Current evidence remains limited, and several factors still constrain clinical and animal studies. First, studies using 18F-FDG PET or PET/CT to assess brain injury after CA remain scarce. Second, tracer development and protocol standardization are important, because FDG uptake can be affected by blood glucose and medications (53), and no broad consensus exists on standardized 18F-FDG PET protocols in this setting (26, 53). The integration of PET-derived metabolic information with MRI, electroencephalography, and serum biomarkers such as NSE and S100β should be evaluated within a multimodal framework. However, this strategy has not yet been tested in large clinical studies. Future prospective studies are needed to validate its incremental value, standardize imaging and interpretation protocols, and determine whether clinical translation after cardiac arrest is justified. Looking ahead, PET-derived metabolic patterns may have three potential clinical research roles in PCABI: treatment guidance, rehabilitation planning, and prognostic assessment. These applications require prospective validation before clinical implementation.

### Real-world clinical limitations and safety considerations

Before routine clinical use in PCABI, 18F-FDG PET/CT must overcome practical and safety barriers that should be weighed against its potential prognostic value. Current evidence remains limited by small cohorts, heterogeneous protocols, and uncertain imaging timing. Therefore, SUV thresholds or regional ratios should not be used as stand-alone prognostic criteria but should instead be interpreted within established multimodal assessment frameworks (26). Cost and access are also important considerations. PET/CT requires scanner availability, radiopharmaceutical production or delivery, trained nuclear medicine personnel, and radiation-protection infrastructure, which may restrict urgent or serial imaging to specialized centers (56). Radiation exposure should be justified, particularly when repeated imaging is considered. For adult brain 18F-FDG PET/CT performed with low-dose CT for attenuation correction or anatomical localization, the approximate effective dose is commonly 4–7 mSv, with the CT component usually contributing less than 0.3 mSv when a low-dose head CT protocol is used. However, the total effective dose may be higher when diagnostic CT, broader scan coverage, higher injected activity, or repeated examinations are used. Therefore, low-dose, locally optimized protocols are required (25, 26, 57). Patients after cardiac arrest often require mechanical ventilation, vasopressor support, temperature control, invasive monitoring, and sedation. Scanner-compatible ICU equipment, including ventilators, monitors, infusion pumps, temperature-management devices, and emergency resuscitation equipment, should be available before PET/CT is performed in critically ill post-cardiac-arrest patients. Experience with PET/CT in ICU patients supports feasibility only when trained personnel, continuous monitoring, emergency equipment, and standardized transport protocols are available. Unstable patients should generally not be transported unless the expected benefit outweighs the risk (58).

Additional confounders are particularly important when interpreting PET/CT after cardiac arrest. Blood glucose should be measured and reported because hyperglycemia and insulin therapy can alter FDG biodistribution and cerebral uptake. Sedatives, anesthetics, antiseizure medications, and neuromuscular blockade should be documented because they may modify cerebral metabolism or mask seizure activity. Mechanical ventilation, vasopressor support, extracorporeal support, temperature-control devices, invasive lines, and infusion pumps require careful transport planning and scanner-compatible monitoring. In practice, 18F-FDG PET/CT should be considered only when the patient is sufficiently stable for transport and when the results are expected to provide information beyond guideline-supported multimodal prognostication (59). Because existing PET studies in PCABI are few, small, and methodologically heterogeneous, no formal head-to-head comparison of PET tracers, PET quantification methods, imaging time points, or PET against established prognostic modalities is currently possible.

## Conclusion

18F-FDG PET or PET/CT provides a biologically plausible approach for visualizing regional cerebral glucose metabolism after cardiac arrest. However, the current evidence base remains too small and heterogeneous to support routine clinical neuroprognostication. Key research priorities include defining optimal imaging windows after ROSC, standardizing acquisition and quantification protocols, systematically reporting confounders related to temperature, sedation, glucose, seizures, and hemodynamics, validating regional and network-level thresholds in multicenter cohorts, and determining whether PET adds incremental value beyond electroencephalography, somatosensory evoked potentials, magnetic resonance imaging, computed tomography, clinical examination, and serum biomarkers. Only after such validation should PET-derived metabolic patterns be evaluated for treatment selection, rehabilitation planning, or long-term outcome prediction. These potential applications should not be interpreted as current clinical recommendations.

## Appendix 1

PubMed/MEDLINE search strategy:((“Positron-Emission Tomography”[MeSH Terms] OR “positron emission tomography”[Title/Abstract] OR “positron-emission tomography”[Title/Abstract] OR “PET/CT”[Title/Abstract] OR “PET-CT”[Title/Abstract] OR “micro-PET”[Title/Abstract] OR microPET[Title/Abstract] OR “Fluorodeoxyglucose F18”[MeSH Terms] OR fluorodeoxyglucose[Title/Abstract] OR FDG[Title/Abstract] OR “18F-FDG”[Title/Abstract] OR “F-18 FDG”[Title/Abstract] OR “FDG-PET”[Title/Abstract] OR “FDG PET”[Title/Abstract] OR “FDG-PET/CT”[Title/Abstract] OR “FDG PET/CT”[Title/Abstract]) AND (“Heart Arrest”[MeSH Terms] OR “Cardiopulmonary Resuscitation”[MeSH Terms] OR “cardiac arrest”[Title/Abstract] OR “heart arrest”[Title/Abstract] OR “cardiopulmonary arrest”[Title/Abstract] OR “cardiorespiratory arrest”[Title/Abstract] OR “cardio-circulatory arrest”[Title/Abstract] OR “circulatory arrest”[Title/Abstract] OR “out-of-hospital cardiac arrest”[Title/Abstract] OR OHCA[Title/Abstract] OR “in-hospital cardiac arrest”[Title/Abstract] OR IHCA[Title/Abstract] OR “post-cardiac arrest”[Title/Abstract] OR “post-cardiac-arrest”[Title/Abstract] OR “post cardiac arrest”[Title/Abstract] OR “post-cardiac arrest syndrome”[Title/Abstract] OR postresuscitation[Title/Abstract] OR “post-resuscitation”[Title/Abstract] OR “post-resuscitation syndrome”[Title/Abstract] OR “return of spontaneous circulation”[Title/Abstract] OR ROSC[Title/Abstract] OR “cardiopulmonary resuscitation”[Title/Abstract] OR CPR[Title/Abstract] OR resuscitated[Title/Abstract])).

Web of Science Core Collection search strategy: TS = ((“positron emission tomography” OR “positron-emission tomography” OR “PET/CT” OR “PET-CT” OR “micro-PET” OR microPET OR fluorodeoxyglucose OR FDG OR “18F-FDG” OR “18F FDG” OR “F-18 FDG” OR “FDG-PET” OR “FDG PET” OR “FDG-PET/CT” OR “FDG PET/CT”) AND (“cardiac arrest” OR “heart arrest” OR “cardiopulmonary arrest” OR “cardiorespiratory arrest” OR “cardio-circulatory arrest” OR “circulatory arrest” OR “out-of-hospital cardiac arrest” OR OHCA OR “in-hospital cardiac arrest” OR IHCA OR “post-cardiac arrest” OR “post-cardiac-arrest” OR “post cardiac arrest” OR “post-cardiac arrest syndrome” OR postresuscitation OR “post-resuscitation” OR “post-resuscitation syndrome” OR “return of spontaneous circulation” OR ROSC OR “cardiopulmonary resuscitation” OR CPR OR resuscitated)).

The final search was conducted in May 2026. Only English-language articles were considered eligible. Publication language was verified during full-text assessment when it could not be reliably determined from the database record at the title and abstract screening stage.

## Glossary

18F-FDG: fluorine-18 fluorodeoxyglucose
ADC: apparent diffusion coefficient
AVS: acute vegetative state
CA: cardiac arrest
CMRglc: cerebral metabolic rate of glucose
CPA: cardiopulmonary arrest
CPR: cardiopulmonary resuscitation
CRS-R: Coma Recovery Scale–Revised
CT: computed tomography
EEG: electroencephalography
ERC–ESICM: European Resuscitation Council–European Society of Intensive Care Medicine
FDG: fluorodeoxyglucose
FIM: Functional Independence Measure
GOS: Glasgow Outcome Scale
GRADE: Grading of Recommendations Assessment Development and Evaluation
GWR: gray–white matter ratio
HIE: hypoxic–ischemic encephalopathy
ICU: intensive care unit
ILCOR: International Liaison Committee on Resuscitation
LCFS: Levels of Cognitive Functioning Scale
MRI: magnetic resonance imaging
NR: not reported
NSE: neuron-specific enolase
OEF: oxygen extraction fraction
OHCA: out-of-hospital cardiac arrest
PCABI: post-cardiac-arrest brain injury
PET: positron emission tomography
PET/CT: positron emission tomography/computed tomography
PVS: persistent vegetative state
ROI: region of interest
ROSC: return of spontaneous circulation
SSEP: somatosensory evoked potentials
SUV: standardized uptake value
SUVmax: maximum standardized uptake value
SUVmean: mean standardized uptake value
SUVR: standardized uptake value ratio
TCD: transcranial Doppler
TLR2: Toll-like receptor 2
TSPO: translocator protein
TTM: targeted temperature management
UCH-L1: ubiquitin C-terminal hydrolase-L1
V/C: vermis-to-cerebellar-hemisphere ratio
VF: ventricular fibrillation
VS: vegetative state
WT: wild type
